# Immune Checkpoint Inhibitor-Induced Endocrine Adverse Events in Cancer Patients at a Tertiary Care Center in Saudi Arabia

**DOI:** 10.7759/cureus.44296

**Published:** 2023-08-28

**Authors:** Ali Alqahtani, Lamia Alghamdi, Abdulmohsen Alghassab, Mussa Almalki

**Affiliations:** 1 Obesity, Endocrine, and Metabolism Center, King Fahad Medical City, Riyadh, SAU

**Keywords:** outcome, immune-related adverse effects, endocrinopathies, cancer, checkpoint inhibitors, immunotherapy

## Abstract

Introduction: Immune checkpoint inhibitors (ICIs) are approved to treat several types of cancer, but they may cause an exaggerated immune response. This can lead to immune-related adverse events such as endocrinopathies, which mostly affect the thyroid and pituitary gland.

Methods: A retrospective analysis was conducted on 125 cancer patients receiving ICIs (pembrolizumab, nivolumab, and ipilimumab) between July 2018 and July 2022. The study reviewed hormone test results and the clinical perspectives of patients to identify and characterize endocrine adverse events associated with ICI therapy in cancer patients.

Results: Among the 125 patients who were examined, a total of 26 patients (20.8%) encountered endocrine-related adverse effects. A total of 25 patients had thyroid dysfunction. Hypophysitis was detected in a limited cohort of two patients, along with primary hypothyroidism. A case of newly diagnosed type 1 diabetes mellitus was seen in a single patient. None of the patients had primary adrenal insufficiency or parathyroid dysfunction. The administration of pembrolizumab was shown to be associated with the occurrence of thyroid dysfunction in 18 cases, as well as two cases of hypophysitis. In contrast, nivolumab was responsible for inducing thyroid dysfunction in four cases. The remaining occurrences were attributable to combination treatment.

Conclusion: The study found an increased risk of thyroid dysfunction among cancer patients receiving ICIs, while pituitary dysfunction was a less frequent adverse effect. It is recommended that an endocrine assessment be conducted before therapy initiation and periodically afterward.

## Introduction

Immune checkpoint inhibitors (ICIs) refer to a class of monoclonal antibody therapies that have been approved for the treatment of various cancers. These therapies function by targeting specific immune checkpoints, namely cytotoxic T-lymphocyte-associated protein 4 (CTLA-4), programmed cell death receptor (PD-1), and programmed cell death ligand 1 (PD-L1) [1].

Typical ICIs include ipilimumab and tremelimumab for CTLA-4 inhibition; nivolumab, pembrolizumab, and cemiplimab for PD-1 inhibition; and atezolizumab, avelumab, and durvalumab for PD-L1 inhibition. The primary mechanism of action involves blocking the inhibitory pathways of T-cell regulation, thereby enhancing the immune system's response to cancerous cells [2]. The immune system has the potential to undergo modifications at two distinct periods. CTLA-4 plays a role in initiating the immune response to antigens during the early stages, whereas PD-1 and PD-L-1 are involved in modulating the interaction between immune cells and peripheral tissues [3].

In 2011, the Food and Drug Administration (FDA) granted approval for ipilimumab, which was the initial ICI drug, specifically for the purpose of treating metastatic melanoma. Subsequently, several other ICI medications have received approval for a range of indications, either as monotherapy or in conjunction with other ICIs or conventional chemotherapy [4,5].

In the past decade, ICIs have profoundly reshaped global oncology treatment approaches, becoming an integral component of various therapeutic regimens for cancer. They have consistently shown potential for enhancing the overall survival rate and prognosis across multiple cancer types. While traditionally employed as adjuvant therapy, there has been a marked shift towards their use as neoadjuvant therapy in recent times. This shift was highlighted in a recent study, which demonstrated that for patients with resectable stage III or IV melanoma, ICIs yielded positive outcomes when administered as neoadjuvant therapy [6]. Nevertheless, these benefits are accompanied by immune-related adverse effects (irAEs) that can vary in severity, potentially leading to fatality [7]. These irAEs arise due to an excessive immune response directed towards non-cancerous cells, resulting in significant inflammation and tissue damage [8].

The systems most impacted by ICIs include the skin, gastrointestinal system, liver, and endocrine system. However, it is important to note that ICIs can affect almost any organ, either during therapy or occasionally several months to years after treatment has been stopped [9]. If properly recognized and addressed, the majority of irAEs can be reversed. However, there is only a small portion of irAEs that are long-lasting or irreversible, requiring the need for long-term replacement medication [10].

The National Cancer Institute created the Common Terminology Criteria for Adverse Events (CTCAE) with the intention of classifying the variety of toxicities, which range from mild (grade 1) to lethal (grade 5). Symptoms classified as grade 3 and higher are considered serious and require hospitalization [11]. The documented occurrence of significant adverse events varies from 26% in cases of monotherapy to 55% in instances where a combination of treatments is employed [12,13]. Endocrinopathy, a commonly observed irAE, has been reported to impact a considerable proportion of patients (about 10%-40%) undergoing ICI therapy. The disorders most observed, listed in descending order, are thyroid disease and hypophysitis [7,14]. Furthermore, it has been observed that the adrenal glands, pancreas, and parathyroid glands are also susceptible to influence [15,16]. A significant proportion of endocrinopathies present with symptoms that lack specificity, hence posing a diagnostic dilemma due to the frequent overlap of these symptoms with those associated with malignancy or further issues arising from therapy. The most reported side effect is fatigue. Consequently, there is a possibility of misattributing symptoms to causes that are less amenable to treatment. The utilization of corticosteroids, antiemetics, and episodes of acute illness associated with immunosuppression serve to complicate the process of diagnosing hormonal axis dysfunction [1]. Symptoms commonly manifest within a six-month timeframe following the initiation of ICI treatment. However, it is important to note that the emergence of symptoms might transpire at any point throughout the therapy or even several months after its discontinuation [15].

It is imperative to promptly identify and effectively manage endocrinopathies caused by ICIs, as failure to do so may lead to severe consequences, including mortality, if left undetected [16,17]. Nevertheless, the current understanding of the safety of ICIs and irAEs remains limited. Consequently, it is imperative to do additional research, such as real-world studies, to supplement randomized controlled trial (RCT) data and bridge existing gaps in our clinical understanding. To the best of our knowledge, our study is the first real-world study to address the endocrine effects of ICIs in Saudi Arabia.

## Materials and methods

Objectives

The purpose of this study is to provide insight into the prevailing adverse medication events linked to ICIs within a single tertiary care center, as well as to distinguish between the adverse events related to different types of ICIs in real-world practice.

Methods 

This single-center retrospective study was conducted at the King Fahad Medical City (KFMC) oncology center, a tertiary referral center. Between July 2018 and July 2022, the hospital electronic system (EPIC) database was searched for patients diagnosed with any type of cancer who received an ICI. Patients were eligible if they were at least 14 years old and had received at least one dose of ICIs. Patients who lacked sufficient documentation were excluded. The medical records of patients were reviewed and analyzed, and the following information was recorded: demographic (age and gender), clinical, radiological, and pathologic data, including tumor type, ICI regimen, treatment response, and survival status. During ICI therapy, all irAEs, including hypophysitis, thyroiditis, adrenalitis, parathyroid disorder, and diabetes, were meticulously recorded. In addition, biochemical parameters, including pituitary hormone, blood glucose, and serum calcium, were recorded at baseline and at follow-up. The study was approved by the institutional review board of the KFMC.

To mitigate potential biases and confounders in our study, we implemented the following strategies: Establishment of clear inclusion and exclusion criteria, adoption of a standardized data extraction process, ensuring consistency and objectivity during the data collection phase, and consideration of the temporal relationship to ascertain a cause-and-effect relationship.

Statistical analysis 

The data that was gathered underwent analysis through the utilization of descriptive statistics. The study patients' demographic and clinical characteristics were presented as mean or median (25th and 75th percentiles) for normally and non-normally distributed continuous variables, respectively. Furthermore, the categorical variables were presented in the form of counts (percentage). The chi-square test was utilized to compare the differences between the two arms. The statistical analyses were conducted using the IBM Corp. Released 2016. IBM SPSS Statistics for Windows, Version 24.0. Armonk, NY: IBM Corp. A significance level of 0.05 was used for all tests, with a two-tailed p-value considered the threshold for statistical significance.

## Results

Results

Baseline Patient Characteristics 

As shown in Table 1, a total of 125 patients were identified and analyzed for the study. The most common cancer was breast cancer (28.0%, n = 35), followed by lung cancer (19.5%, n = 24), metastatic renal cell carcinoma (8.9%, n = 11), and nasopharyngeal cancer (5.7%, n = 7). The mean age at initiation of ICI was 53.7 ± 14.9 years. There were 69 females, constituting 55% of the total population. During the time covered by this study, the average body mass index (BMI) was 26.17 6.1 kg/m2. There was no correlation found between BMI and age regarding symptoms or the severity of endocrine dysfunction. Most patients (91.2%, n = 114) were treated with monotherapy, whereas a minority (8.8%, n = 11) received a combination of therapeutic agents. Most patients (76.8%, n = 96) were treated with pembrolizumab, followed by nivolumab (26.4%, n = 33) and ipilimumab (5.7%, n = 7). A combination of pembrolizumab or nivolumab with ipilimumab was administered to 8.8% of patients (n = 11), as shown in Table 2. The dose of pembrolizumab was 200 mg intravenously every three weeks, while ipilimumab was given at a dose of 240 mg intravenously every two weeks. The average number of cycles administered was 12, and the average treatment lasted 12 months.

**Table 1:** Baseline population characteristics

Characteristics	Mean (SD)	No. (%)
Age	53.73 (14.9)	
BMI	26.17 (6.1)	
Gender		
Male		56 (44.8)
Female		69 (55.2)
Type of cancer		
Breast cancer		35 (28)
Lung cancer		24 (19.5)
Metastatic RCC		11 (8.9)
Nasopharyngeal cancer		7 (5.7)
Endometrial cancer		6 (4.9)
Cervical cancer		5 (4.1)
Mandibular cancer		4 (3.3)
Hodgkin lymphoma		6 (4.9)
Gastric cancer		4 (3.3)
Other types of cancer		23 (18.4)
Number of agents used		
One agent		114 (91.2)
Combination (two agents)		11 (8.8)
Agent used		
Pembrolizumab		96 (76.8)
Nivolumab		33 (26.4)
Ipilimumab		7 (5.7)
Number of treatment cycles	11.54 (10.9)	
Duration of treatment (months)	11.98 (10.35)	

**Table 2:** Endocrine-related adverse effects according to ICI type

	n (%)
Primary thyroid dysfunction	
Pembrolizumab	19 (76%)
Nivolumab	4 (17)
Combination therapy (Pembrolizumab or Nivolumab + ipilimumab)	2 (8%)
Hypophysitis	
Pembrolizumab	2 (1.6 %)
Diabetes mellitus	
Nivolumab	1 (0.8%)

Endocrine-related adverse effects (ERAE), incidence, and characterization 

A total of 26 (20.8%) patients experienced endocrine-related adverse effects over the mean treatment duration of 12 months, as presented in Table 3. The most frequent endocrine disease was thyroid dysfunction, which was reported in 20% (n = 25) of patients who received ICI. Hypophysitis developed in two (1.6%) patients, along with primary hypothyroidism. The ACTH deficiency was the most and only affected axis. New-onset type 1 diabetes mellites appeared in one patient.

**Table 3:** Endocrine-related adverse effects

ERAE development	n (%)
Primary thyroid dysfunction	25 (20)
Overt hypothyroidism	17 (68)
Subclinical hypothyroidism	7 (29)
Thyroiditis	1 (4.2)
Thyrotoxicosis	0 (0)
Hypophysitis (Corticotroph axis only)	2 (1.6)
Diabetes	1 (0.8)
Primary adrenal insufficiency	0 (0)
Parathyroid disorder	0 (0)

There was variability in the timing of testing in relation to the start of ICIs due to the absence of a standardized testing procedure for screening potential endocrine adverse effects within our cohort. As a result, the time needed for the onset of endocrine dysfunction, particularly thyroid dysfunction, cannot be determined precisely in this context.

Thyroid dysfunction occurred in 25 patients. Most patients were frankly hypothyroid (68%, n = 17), subclinically hypothyroid (29%, n = 7), or remained euthyroid with a clinical and radiological picture of thyroiditis (4.2%, n = 1). Nineteen (76%) patients were treated with anti-PD-1 (pembrolizumab), four (17%) with anti-PD-1 (Nivolumab), and two (8%) with combination immunotherapy developed primary thyroid abnormalities. Individuals who developed hypothyroidism and symptomatic sub-clinical hypothyroidism were only commenced on thyroxin replacement. The prevalence of thyroid dysfunction was found to be higher in patients diagnosed with cervical cancer (40%) as compared to other cancer types, such as renal cell cancer (27.3%), lung cancer (20.8%), and breast cancer (20.6%). The patients who experienced thyroid dysfunction were administered a greater number of treatment cycles (with a mean of 14.8 cycles) and were subjected to a longer duration of treatment (with a mean of 14.2 months) as compared to those who did not develop endocrinopathy. The latter group received 10.8 cycles and 11.4 months of treatment, respectively.

Two patients (60-year-old female and 33-year-old female) were diagnosed with hypophysitis in association with anti-PD-1 (pembrolizumab) use for breast cancer. Both presented symptomatically with a combination of fatigue, myalgia, nausea, and visual changes in one of them. The initial hormonal evaluation was suggestive of secondary adrenal insufficiency, which was subsequently confirmed by an ACTH stimulation test while another pituitary axis was preserved. MRI pituitary revealed globular pituitary enlargement, for which they were taken off Pembrolizumab and began treatment with physiologic doses of steroid replacement (hydrocortisone 10 mg a.m. and 5 mg p.m.) and thyroid replacement for primary hypothyroidism as per standard clinical practice. Repeated MRI pituitary during subsequent follow-up (8-10) months showed complete normalization of the pituitary enlargement.

Diabetes mellitus was a pre-existing condition in 24.4% of all patients, with approximately 4% receiving insulin treatment. A newly diagnosed diabetes was reported in one old female patient who had metastatic HCC with liver cirrhosis and presented with diabetic ketoacidosis one month after the commencement of immunotherapy with Nivolumab. Further biochemical evaluation revealed glycated hemoglobin (HbA1c) of 7.7% and very low C-peptide < 10 pmol/l (260.00-1,730.00), while anti-glutamate decarboxylase antibodies and islet cell antibodies have not yet been detected. The patient initially received treatment with intravenous fluids and an insulin infusion, then subsequently transitioned to subcutaneous insulin. Despite discontinuing immunotherapy, the patient remained insulin-dependent throughout the six-month follow-up period.

None of the patients developed primary adrenal insufficiency or parathyroid disorders. The survival rate was not recorded due to a lack of mortality data. No detailed data for mortality was available for this study. Most patients either had no symptoms or had non-specific symptoms. Apart from two patients who presented with visual symptoms and symptoms of adrenal insufficiency and were later diagnosed with hypophysitis, the detection of these patients was entirely attributed to screening tests.

## Discussion

The findings of this study indicate that a significant proportion, approximately 20.85%, of patients undergoing immunotherapy treatment experience the development of endocrinopathies. This prevalence aligns with the range of rates reported in previous studies [18,19]. The precise onset time of endocrinopathies could not be determined in this study due to variations in the timing of tests relative to the initiation of ICIs. Prior research has indicated that the onset of endocrinopathies occurs within a timeframe of 9 to 12 weeks following the administration of CTLA-4 and within an interval of 4 to 18 weeks following the administration of anti-PD-1 [20,21]. This data is of utmost importance, as it carries significant implications for the clinical management and duration of patient surveillance. 

In contrast to the findings of previous studies [21], the patients in our cohort who underwent monotherapy treatment with pembrolizumab reported a greater occurrence of endocrine immune adverse events compared to those who received combination therapy. The disparity in the occurrence of endocrine immune-related adverse events between monotherapy and combination therapy is most likely due to a small sample size and the low number of patients undergoing combination therapy.

Thyroid dysfunction is one of the most frequent adverse effects associated with ICI therapy [22,23]. It is believed to be predominantly associated with anti-PD-1 therapy as well as anti-PD-1 and anti-CTLA-4 combination therapy [24]. Most patients in this study (n = 26) were diagnosed with thyroid dysfunction. Seventy-five percent of the thyroid toxicity associated with PD-1 was caused by pembrolizumab, while 4% was induced by nivolumab. This is consistent with the existing medical literature, which indicates that anti-PD-1 and anti-PD-L1 therapies carry a higher risk than anti-CTLA4 agents, with the greatest risk reported for combination therapy [25]. Moreover, our study revealed that hypothyroidism was reported significantly more often than hyperthyroidism (16 vs. 0), which is consistent with the findings of the clinical study [26]. The estimated incidence of hypothyroidism is 2.5%-3.8% (anti-CTLA4), 3.9%-8.5% (anti-PD1/PDL1), and 10.2%-16.4% (combination therapy) [19]. Conversely, the occurrence of thyrotoxicosis is reported to be lower, with incidence rates ranging from 0.2%-5.2% for anti-CTLA4 therapy, 0.6%-3.7% for anti-PD-1/PDL1 therapy, and between 8% and 11.1% for combination therapy [19].

Except for patients who concurrently experienced hypophysitis, none of the patients who encountered adverse reactions from their immunotherapies discontinued their treatment. Hypophysitis exhibits a higher incidence rate among patients undergoing anti-CTLA-4 therapy, with the potential to affect approximately 10% of the patient’s population [26,27]. The association between anti-PD-1, PD-L1 therapy, and hypophysitis has also been documented. A total of 276 cases of hypophysitis have been reported in the medical literature between 2003 and 2019. Among these cases, 70% were associated with CTLA-4 blockade, 23% with PD-1 blockade, 2% with PD-L1 blockade, and 3.9% with combination therapy involving both CTLA-4 and PD-L1 inhibitors [28].

The findings of our study indicate a lower incidence of hypophysitis (n-2) following the administration of the fourth cycle of treatment with pembrolizumab (PD-1). Considering the estimated incidence of 1%-5% [29,30], it is not surprising that hypophysitis is an infrequent complication of ICI therapy. However, another study found a higher incidence [31], which may be attributed to the implementation of universal routine screening, resulting in earlier detection of early-onset hypophysitis. The patients in our study were found to have ACTH deficiency, a condition that aligns with findings from previous research. These studies have consistently shown that secondary adrenal deficiency is a prevalent hormone deficiency, occurring in 83% of cases. Additionally, secondary hypothyroidism and hypogonadotrophic hypogonadism are reported in 77% and 55% of cases, respectively [24,32,33].

It is common for patients to present with multiple hormone deficiencies, with three deficiencies being the most frequently observed [29]. The prevalence of growth hormone (GH) deficiency remains uncertain, primarily because routine dynamic testing for the GH axis is not commonly performed. Upon subsequent examination, the pituitary abnormalities depicted in the images exhibited complete normalization. According to a previous study, it has been found that a significant proportion of cases resulting from anti-CTLA-4 treatment exhibit pituitary MRI abnormalities in 81% of cases. Similarly, patients with hypophysitis who undergo treatment with anti-PD-1/anti-PD-L experience such abnormalities in 18% of cases. Notably, these abnormalities manifest as an initial enlargement of the pituitary gland, which subsequently reverts to its normal size within a few weeks [34-36].

The reported incidence of ICI-induced diabetes varies between 0.9 and 2% [28], with a significant proportion of cases, up to 76%, attributed to the use of anti-PD-1 agents, 8% to anti-PD-L1 agents, and only 4% to the use of anti-CTLA-4 agents [7,37]. It is worth mentioning that type 1 diabetes (T1D) induced by ICI has been documented in relation to all PD-1 inhibitors currently in clinical use, such as pembrolizumab and PD-L1 inhibitors. However, occurrences of ICI-induced T1D with CTLA-4 inhibitors like ipilimumab are relatively rare [38]. In our study, we observed the occurrence of new-onset type 1 diabetes mellitus in a single patient who presented with diabetic ketoacidosis (DKA). This could be attributed to the limited sample size or variations in patient demographics, which include a substantial proportion of pre-existing diabetes mellitus cases (24.4%) in our study group. The results of our study align with existing medical literature, which indicates that most ICIs have been found to induce type 1 diabetes mellitus (T1DM) in elderly patients, predominantly reported in cases involving the use of anti-PD-1 therapies such as nivolumab and pembrolizumab [38].

Notably, the present study did not describe any cases of parathyroid-related adverse effects, which is consistent with the low rates of such events described in existing literature, with only five cases of hypoparathyroidism reported in the literature [39,40]. In a recent study involving a cohort of 178 patients who underwent treatment with immune checkpoint inhibitors (ICI), the occurrence of true hypocalcemia was observed in only one case [41]. Nevertheless, there have been documented cases of hypercalcemia in patients receiving ICIs, although it appears to be non-PTH-mediated [28]. The occurrence of adrenal insufficiency has been infrequently documented in the literature. Recent studies have reported a prevalence of approximately 1% in cases of monotherapy and a higher prevalence ranging from 5%-7% in cases of combination therapy [11,18]. Likewise, no cases were detected within our cohort. In our study, pembrolizumab has been identified as the most frequently implicated ICI in endocrine-related adverse events. This finding is consistent with the results reported in previous studies [42,43].

It is important to recognize that the use of pembrolizumab as an ICI was widespread in our study population, which may have influenced the observation mentioned earlier. The relationship between the type of cancer and the probability of encountering adverse events related to the endocrine system is currently not well understood, and additional investigation is required to gain a better understanding of the underlying mechanisms [44]. In our study, it was observed that cervical cancer exhibited a greater incidence of thyroid dysfunction (40%) in comparison to other forms of cancer, namely renal cell cancer (27.3%), lung cancer (20.8%), and breast cancer (20.6%). Nevertheless, it is crucial to consider the restricted number of patients involved in the study and the possibility of other variables that may have influenced the outcomes, as these factors could impact the interpretation of the findings.

The present study possesses several noteworthy limitations that necessitate acknowledgment. These limitations include the retrospective design employed, the absence of a standardized testing protocol for assessing endocrine adverse effects, the relatively small sample size, and the fact that the study was conducted at a single center. The generalizability of our results may be hindered by the limitations of our findings and the difficulties in identifying rare endocrine-related adverse events.

## Conclusions

Immunotherapy treatments offer substantial benefits to individuals diagnosed with advanced cancer. Despite the notable adverse effects associated with these therapies, the heightened rates of survival they offer serve to rationalize the associated risks. The findings of our study indicate a significant occurrence of endocrine-related adverse effects among cancer patients who received immunotherapy with ICIs. Thyroid dysfunction emerged as the most observed adverse event. Pembrolizumab emerged as the predominant ICI employed, and it was identified as the primary etiological factor for thyroid dysfunction and hypophysitis. The findings of our study underscore the importance of diligent monitoring of endocrine function in patients receiving ICIs. Additionally, our study highlights the importance of establishing standardized screening protocols to detect potential negative consequences. The prompt recognition and effective management of complications associated with the endocrine system possess the capacity to alleviate their detrimental effects on patient outcomes and lifespan. Additional study is required to enhance comprehension of the variables that contribute to vulnerability and resilience, as well as approaches for maximizing existing interventions.
